# Vocal Motor Performance in Birdsong Requires Brain–Body Interaction

**DOI:** 10.1523/ENEURO.0053-19.2019

**Published:** 2019-06-25

**Authors:** Iris Adam, Coen P. H. Elemans

**Affiliations:** Department of Biology, University of Southern Denmark, 5230 Odense, Denmark

**Keywords:** development, motor control, muscle training, sensorimotor learning, songbird

## Significance Statement

Motor skill learning typically occurs in a period when the brain needs to navigate a body that is still growing and developing. How the changing body, neural circuit formation, and motor coding influence each other remains unknown. Songbirds provide excellent model systems to study motor skill learning. It has recently been shown that songbird vocal muscles double in speed during sensorimotor learning. Here we argue that these contractile as well as morphological changes stem predominantly from use and only secondarily from hormones or genetic programs. This implies that muscle training constrains skill-learning trajectories. As contractile muscle property changes must require altered motor codes for achieving the same acoustic targets, the final performance results from interactions between brain and body.

## Introduction

Understanding how novel behaviors are learned remains a major challenge to modern neuroscience. Acquiring and mastering fine motor skills, from dexterity in piano playing to microsurgery or speech, can take weeks to months or even years and is strongly affected by injury, stroke, and developmental as well as neurodegenerative disorders. Most fine motor skill learning occurs postnatally from infancy to adolescence when the brain needs to navigate a body that is exhibiting large changes due to growth and development. Changes in neural coding and circuit development remain challenging to follow over meaningful timescales in single individuals and are thus typically studied during rather brief periods in adult subjects (Li et al., 2001; Xiao et al., 2006) or during recovery after injury (Nudo et al., 1996; Dancause et al., 2005). Songbirds however, provide a powerful and unique system to study motor skill learning over ethologically meaningful time scales (Brainard and Doupe, 2013).

The brain does not function in isolation. All animal behaviors result from complex system-wide interactions between nervous system, body, and surrounding environment (Chiel and Beer, 1997; Lum et al., 2005; Nishikawa et al., 2007; Tytell et al., 2011; Düring and Elemans, 2016; Sober et al., 2018). Motor pathways produce precisely timed complex sequences of motor commands to activate muscles. The forces ultimately generated by muscles strongly depend on dynamic body motion and environmental conditions through the muscle’s nonlinear force–length and force–velocity properties (Düring and Elemans, 2016). Thus, motor control systems are closed-loop systems (Roth et al., 2014) and the activity of neural circuits can be understood only by considering the biomechanics of muscles, bodies, and the exterior world (Tytell et al., 2011).

How the developing body influences circuit formation and neural coding in the brain and vice versa is still largely unknown (Avitan and Goodhill, 2018). Recent work showed that developmental changes in vocal behavior of marmoset monkeys that were typically attributed to neural changes can be explained by changes in the body (i.e., growth of the lungs; Zhang and Ghazanfar, 2018), emphasizing the need for an embodied view on motor control during vocal development.

In this opinion piece, we argue that contractile changes occurring in the vocal muscles of songbirds during song learning stem predominantly from interactions between brain and body. This implies that extensive training of syringeal muscles is essential to achieve their maximal performance and that the duration and trajectory of song learning are not solely set by neural circuit formation. Given that virtually all motor skills or at least their building blocks are acquired during times while the body is still changing, truly understanding motor coding and its pathologies requires rethinking and an embodied approach to understand motor learning.

## Songbird brain and body change over vocal development

The sensorimotor phase of song learning in zebra finches takes ∼2 months and starts when juveniles start producing subsong at 28 d post-hatching (DPH). Song development proceeds from subsong through plastic song to adult, so-called crystallized song, which is reached ∼100 DPH (Roper and Zann, 2006). Over the course of vocal development, a network of discrete interconnected forebrain nuclei dedicated to learn and produce song (hereafter referred to as song system) is changing profoundly in morphology and function. Significant research effort has been dedicated to describing and understanding learning related changes in the brain during vocal development in songbirds (Fee and Goldberg, 2011; Brainard and Doupe, 2013). However, despite our rapidly advancing knowledge of the song system, unfortunately very little is known about the motor code that drives the three main motor systems involved in sound production: the vocal organ, the syrinx; the respiratory system; and upper vocal tract (Elemans, 2014; Schmidt and Goller, 2016). The motor neurons that control all these muscles are all located in small oblong nuclei in the brainstem (Schmidt and Martin Wild, 2014) and their location and small size complicates chronic recording in freely behaving animals even in adults (Williams and Nottebohm, 1985). At the terminus of the premotor pathway of the song system, *in vivo* recordings show that over vocal development the premotor neurons gradually change their firing pattern from highly variable patterns into sparse high-frequency bursts (Ölveczky et al., 2011). In adult males these premotor neurons are precisely locked to song timing (Sober et al., 2008; Ölveczky et al., 2011) and can causally explain variation in biomechanics and behavior of the respiratory motor system (Srivastava et al., 2017).

In parallel, the syrinx also exhibits changes during the sensorimotor learning phase. Syringeal muscle mass and cross-sectional area increase from hatching to adulthood and sex differences can be found after 20 DPH in zebra finches (Godsave et al., 2002). However, such morphological changes do not allow reliable inferences about changes in contractile properties of the muscle, such as contraction speed, maximal tension, force-length, and force-velocity profiles. Because contractile muscle properties determine the forces that act on body and environment, they are critical traits for understanding the biomechanics of vocal production and thereby linking motor commands to behavioral output, i.e., song. It has recently been shown that over song learning, the superfast syringeal muscles controlling song double in isometric contraction speed and ultimately reach the maximal attainable speed possible in vertebrate synchronous muscle (Mead et al., 2017). The muscle speed increase was associated with a composition change of expressed heavy myosin chain gene isoforms (*MYH*) toward near-exclusive expression of *MYH13* aka superfast myosin (Mead et al., 2017). Concluding, over vocal development syringeal muscles exhibit changes in morphology as well as contractile properties.

In the following sections we will review the three most likely factors that could drive these changes: hormones, an innate developmental program, or neural drive.

## Hormonally mediated changes cannot explain adult syrinx dimorphism

In adult zebra finches, the entire song system, including the syrinx, exhibits differences between sexes. Most nuclei of the song system are smaller in females (Nixdorf-Bergweiler, 1996; Shaughnessy et al., 2019), females have a smaller motor nucleus projecting to the syrinx (Godsave et al., 2002; Wade et al., 2002), the syrinx itself is also smaller with a less developed skeleton (Düring et al., 2013), and syringeal muscles have less mass (Bleisch et al., 1984; Wade and Buhlman, 2000; Düring et al., 2013) and a lower contraction speed (Elemans et al., 2008), albeit still nearly two orders of magnitude faster than locomotory muscles. Moreover, syrinx muscles in females express less *MYH13* than in males (Mead et al., 2017). The sexual dimorphic nature of these observations suggests that these traits are mediated by gonadal steroid hormones.

In general, this hypothesis is supported by previous studies showing that the syrinx is sensitive to manipulations of steroid hormone levels: treating adult females with testosterone increases muscle mass, fiber diameter, and number of acetylcholine receptors, masculinizing the syrinx (Bleisch et al., 1984; Wade and Buhlman, 2000). Similarly, decreasing testosterone levels in males feminizes the syrinx, which is reflected in a loss of muscle mass (Luine et al., 1980; Bleisch et al., 1984; Wade and Buhlman, 2000). However, masculinization never reaches male levels and axotomizing (severing the innervating nerve) the syrinx of testosterone-treated females abolishes masculinization (Bleisch et al., 1984; Wade and Buhlman, 2000), which indicates that sex differences in syringeal properties are not solely driven by steroid hormones. This is further supported by the finding that androgen receptor expression only gets dimorphic after 30 DPH, 10 d after muscle weight and cross-sectional-area start to be different between the sexes (Godsave et al., 2002). Thus, the changes in muscle morphology seem to be driven by the change in neural drive because of the onset of singing activity in males ∼28 DPH (Arnold, 1975) rather than direct action of hormones on the syrinx. This hypothesis is further strengthened by the finding that castration changes the normal song learning trajectory only slightly, mostly by decreasing the amount of song per day and delaying crystallization in some but not all animals (Arnold, 1975).

Together we conclude that steroid hormones are critical but not sufficient to induce and maintain sexual dimorphism of the syrinx.

## Muscle use drives morphological and contractile changes

Skeletal muscle functionality is known to change with use in adults or after peripheral nerve damage and use reduction causes muscle atrophy (Buller et al., 1960, 1987; Lømo, 2003; Schiaffino and Reggiani, 2011). As such, the most drastic experiment to investigate neural drive on muscles is axotomy, i.e., to severe the nerve innervating the muscle. Axotomizing the syrinx in songbirds leads to a decrease in fundamental frequency and amplitude of the produced sound and the conversion of all syllables into harmonic stacks because of the loss of fast frequency modulations (Williams et al., 1992; Daley and Goller, 2004; Roy and Mooney, 2007; Secora et al., 2012; Vallentin and Long, 2015). Morphologically, syringeal muscles atrophy after axotomy (Urbano et al., 2013), suggesting that usage is required to maintain muscle mass. However, it remains unknown how axotomy affects contraction related properties, such as contraction speed, force-length and force-velocity profiles, *MYH* composition, and *MYH13* expression in particular.

The discovery of *MYH13* expression in syringeal muscles (Mead et al., 2017) placed them conclusively in the lineage of craniofacial muscles, corroborating earlier developmental studies (Noden et al., 1999; Noden and Francis-West, 2006). These skeletal muscles can uniquely express several rare myosin heavy chain isoforms, among them *MYH13*. The craniofacial muscles also include extraocular and most laryngeal muscles (Briggs and Schachat, 2000; Hoh, 2010) and are characterized by extremely fast force development, reaching maximal tension within a few milliseconds (Elemans et al., 2008; McLoon and Andrade, 2012). *MYH13* has been proposed to be responsible for very high contraction speeds and this notion is strengthened by the unusually high detachment rate from actin (Bloemink et al., 2013).

In our opinion, the most likely hypothesis to explain the upregulation of *MYH13* and doubling in contraction speed in the syrinx during song learning is that the increased use, or training, of muscles is causally driving these changes. This hypothesis is supported by three lines of evidence:

1. *Timescale of upregulation.* In all craniofacial muscles studied to date that express myosin *MYH13*, contraction speed as well as *MYH13* expression increase over a similar, relatively slow (i.e., weeks) time course after birth. In songbirds, *MYH13* expression changes over 2 months during sensorimotor learning (Mead et al., 2017). In kittens, twitch speed as well as tetanic tension of extraocular muscles increases during the first 20 weeks of life (Lennerstrand and Hanson, 1978). In mouse and rat extraocular muscles and the rat larynx *MYH13* expression increases over the first 20–30 d of life (Shiotani et al., 1999; Zhou et al., 2010; Moncman et al., 2011), but to our knowledge no data on the development of contraction speed is available in these muscles.
2. *Neural drive influences muscle speed and MYH expression.* In general, skeletal muscle functionality changes with neural drive. Use reduction causes muscle atrophy (Buller et al., 1960, 1987; Lømo, 2003; Schiaffino and Reggiani, 2011) and transnervation with a faster spiking nerve increases contraction speed (Paniello et al., 2001). Neural stimulation is known to drive *MYH* expression patterns and contraction speed: in rats, neural activity associated with optokinetic and vestibulo-ocular reflexes stimulates *MYH13* expression in extraocular muscles (Brueckner et al., 1999; Moncman et al., 2011). In kittens, the earlier described postnatal increase in contraction speed is impeded by impairing binocular vision (Lennerstrand, 1979; Lennerstrand and Hanson, 1979). Likewise, upregulation of *MYH13* in rodent extraocular and laryngeal muscles is prevented by visual deprivation or axotomizing the larynx, respectively (Brueckner and Porter, 1998; Shiotani and Flint, 1998; Brueckner et al., 1999), which in addition provides evidence against the hypothesis that a fixed postnatal developmental program controls *MYH13* upregulation. Last, in all *MYH13* expressing craniofacial muscles, the increase in contraction speed and *MYH13* expression coincides with the onset of muscle use (Lennerstrand and Hanson, 1978; Shiotani et al., 1999; Zhou et al., 2010; Moncman et al., 2011; Mead et al., 2017).
3. *Location of MYH13 transcription.* In particular the expression of *MYH13* opens the possibility that changes in the properties of syringeal muscles are driven by neural activity, as *MYH13* is known to be transcribed close to the neuromuscular junction in extraocular muscles (Briggs and Schachat, 2002). It has been speculated that the electrical or chemical activation of motor neurons directly stimulates *MYH13* transcription linked to acetylcholine receptors (Sanes and Lichtman, 2001; Rubinstein et al., 2004). However, it is unknown to what extent the amount of stimulation affects *MYH13* transcription and other muscle properties.

## Conclusions and implications

Together the data above strongly suggest that the postnatal increase in muscle speed and *MYH13* expression in the songbird syrinx is primarily caused by the use of the muscles and only secondarily because of hormones and innate genetic programs.

We propose this hypothesis has several implications for motor learning:

1. **Time course of song learning is set by muscle training**. Because previous work established that *MYH13* expression is regulated by use and correlates to speed, we suggest that extensive use of syringeal muscles is essential to achieve their maximal speed. The timescale of *MYH13* upregulation in craniofacial muscles is >4 weeks in all studied model systems, and ∼7 weeks in songbirds. Because this duration overlaps the typical temporal trajectory for skeletal muscle endurance or speed training, we speculate that the total duration and trajectory of song learning is not solely set by neural circuit formation but must also be required for muscle training. As female songbirds may be able to perceive millisecond scale variations (Prior et al., 2018) and prefer sped-up song in several species (Drăgănoiu et al., 2002; Weiss et al., 2012), we further speculate that millisecond fine-scale acoustic modulations, such as frequency modulation or on- and offset precision, can act as an honest signal (Searcy and Nowicki, 2005) for metabolic energy invested in muscle training.
2. **Execution error is constrained by immature motor code and muscle speed**. In adults, premotor codes and song behavior occur at millisecond scale precision (Chi and Margoliash, 2001; Hahnloser et al., 2002) and recently variation in spike timing at millisecond timescales has been shown to causally affect biomechanics and behavior (Tang et al., 2014; Srivastava et al., 2017; Sober et al., 2018). In contrast, the plastic phase of song learning is marked by a higher rendition to rendition variability in acoustic parameters like syllable duration and frequency (Ali et al., 2013). This variability is essential for vocal exploration during trial and error learning (Charlesworth et al., 2011) and thought to be driven mostly by the basal ganglia (Kojima et al., 2018). We propose an additional interpretation: faster muscles allow a more temporally precise actuation to achieve acoustic targets leading to less variability in, e.g., duration of syllables. In other words, we assume that muscle speed increase leads to execution error decrease. We hypothesize that the (initially) slower superfast syringeal muscles constrain the precision to execute motor sequences during vocal learning, especially at the start of song ontogeny. We predict that variable vocalizations observed during song learning reflect immature brain circuits as well as muscle speed.
3. **Changes in contraction speed require motor code adaptation for achieving the same target.** During motor learning, animals try to minimize error for achieving task-specific motor targets. In the zebra finch, the acoustic song template is considered to not change during sensorimotor learning (Mooney, 2009). As contraction speed of syringeal muscles changes over the course of weeks, playing a hypothetical stereotyped motor code will lead to changes in force profiles. Consequently, the acoustic targets, such as amplitude, entropy, or fundamental frequency, will also change and in turn lead to changes in error magnitude. Thus, we propose that the observed contractile changes of syringeal muscles require altering the motor code during development to achieve the same force profiles and acoustic targets.

The hypotheses presented above remain to be tested experimentally. Only with a systems view will we be able to explain and understand the development of complex behaviors. Given the dedicated neural, muscular, and genetic substrates, the knowledge accrued to date and the increasing number of genetic tools (from RNA-interference to DREADDs and optogenetics) available to the field, birdsong is an ideal system to embrace such an integrative approach.

## Synthesis

Reviewing Editor: Darcy Kelley, Columbia University Decisions are customarily a result of the Reviewing Editor and the peer reviewers coming together and discussing their recommendations until a consensus is reached. When revisions are invited, a fact-based synthesis statement explaining their decision and outlining what is needed to prepare a revision will be listed below. The following reviewer(s) agreed to reveal their identity: Arthur Leblois, Richard Hahnloser.

Birdsong is a classical paradigm used to study skill learning. Large changes undergone by vocal muscles during the song acquisition process represent a challenge for the neural system driving learning and illustrate the feedback interactions between brain and body that occur during skill learning. In this opinion, the authors show that functional changes in the vocal muscles of juvenile songbirds occur predominantly due to muscle use, rather than following an innate program. This implies that the motor code being learned is constantly changing during the song learning process, changing our view on the learning process and its neural substrate. The opinion piece is well written and reflects well the current knowledge on this topic. It presents clear hypothesis and conclusions. One general point remains unclear: the authors mention both functional changes in muscles (muscle speed and force) and morphological changes (size, weight). In the abstract and conclusion, they clearly link the use of muscle to functional changes. However, in the text they also refer to morphology. In particular, they claim that hormonal signaling changes are too late to induce morphological changes (line 123). Muscle use in juvenile males starts even later (day 35) and cannot be causal to those changes either. The logic of this part and its articulation to the rest of the paper should be improved. This review also makes an interesting point about heavy myosin chain gene (MYH) and its potential role in enabling adult song features in songbirds. However, the manuscript overall is difficult to read as the authors discuss too many topics too densely and in a confusing order and would be much strengthened by focusing on just the main points and thoroughly discussing these.

Title: The title is too vague and sounds a bit like a triviality. One of the main points of the abstract, use of muscles vs aging, is not clearly emphasized in the paper, is this the main point?

Introduction: The introduction takes too long to get to the main point, the entire first page is about motivating songbirds whereas it should be about muscles and the role of MYH during development (including monkeys, cats, and rats which are first mentioned only several pages later). Authors should more thoroughly discuss the need for new experiments (e.g. on the role of MYH in isolate birds).

Specific comments:

line 49 remove full stop.

lines 46-48: the end of this sentence is not clear

lines 70-75: the general statements about previous research about the neural substrate of song learning are a bit simplistic and partial. It would be more accurate to cite recent reviews about the song system and its functions, rather than a sparse (and arbitrary) selection of a few research papers from the last 25 years.

line 77: when does syringeal muscle mass and cross-sectional area increase?

line 82: comma after learning.

line 85: three times precise.

line 86: citing Hahnloser 2002 for precise motor code is not the most accurate, as HVC code is not really motor, rather temporal. For precision of motor code in RA, see papers by the group of Sober.

line 87: New paragraph. Also accord tenses in the intro: past tense instead of present.

line 107: comma missing.

line 133: axotomy should be discussed in more depth, effects on motor changes must be expected to be profound.

line 138: vestibulo

line 141: what is X with XII?

line 144: activates motor neurons

line 149: birth is slow?

lines 166-190: In the paragraph ‘conclusions’, the authors mix statements that are real conclusion from the literature and speculations (ex: point 2, authors recognize that it's a speculation). The speculation and conclusions should be clearly separated and presented as such. Also, some conclusions are overlapping and the paragraph could be clarified

line 167: maximal performance of what? citation?

line 172: temporal trajectory

line 178: comma after vocal learning

line 182: comma after Recently

line 187: remove ‘try to’
